# Protein Turnover in Mycobacterial Proteomics

**DOI:** 10.3390/molecules14093237

**Published:** 2009-08-28

**Authors:** Prahlad K. Rao, Qingbo Li

**Affiliations:** 1 Center for Pharmaceutical Biotechnology, College of Pharmacy, University of Illinois at Chicago, Chicago, Illinois 60607, USA; 2 Department of Microbiology and Immunology, College of Medicine, University of Illinois at Chicago, Chicago, Illinois 60612, USA

**Keywords:** *Mycobacterium tuberculosis*, stress response, protein turnover, proteome dynamics

## Abstract

Understanding the biology of *Mycobacterium tuberculosis* is one of the primary challenges in current tuberculosis research. Investigation of mycobacterial biology using the systems biology approach has deciphered much information with regard to the bacilli and tuberculosis pathogenesis. The modulation of its environment and the ability to enter a dormant phase are the hallmarks of *M. tuberculosis*. Until now, proteome studies have been able to understand much about the role of various proteins, mostly in growing *M. tuberculosis* cells. It has been difficult to study dormant *M. tuberculosis* by conventional proteomic techniques with very few proteins being found to be differentially expressed. Discrepancy between proteome and transcriptome studies lead to the conclusion that a certain aspect of the mycobacterial proteome is not being explored. Analysis of protein turnover may be the answer to this dilemma. This review, while giving a gist of the proteome response of mycobacteria to various stresses, analyzes the data obtained from abundance studies *versus* data from protein turnover studies in *M. tuberculosis*. This review brings forth the point that protein turnover analysis is capable of discerning more subtle changes in protein synthesis, degradation, and secretion activities. Thus, turnover studies could be incorporated to provide a more in-depth view into the proteome, especially in dormant or persistent cells. Turnover analysis might prove helpful in drug discovery and a better understanding of the dynamic nature of the proteome of mycobacteria.

## 1. Introduction

Tuberculosis is one of those diseases that has yet to relinquish its grip over mankind [1]. Complete eradication of tuberculosis is still not anticipated in the foreseeable future [2]. The problem resides in the ability of *M. tuberculosis* to persist in a presumably dormant state [3]. Thus, persistence contributes to a latent tuberculosis infection which serves as an enormous reservoir of infection [4,5]. According to the WHO, 184 nations have adopted the Directly Observed Therapy Short Course treatment regimen for their national tuberculosis control programs [6]. But the long duration of these regimens has made it difficult to maintain compliance in many areas. As a consequence, non-compliance contributes to the emergence of multidrug-resistant and extremely-drug-resistant *M. tuberculosis* strains that impose an even greater threat. Currently, multidrug-resistant tuberculosis infections have a mortality rate >50% and their cure requires a 2-year course of expensive and highly toxic treatments. The extremely-drug-resistant tuberculosis infection is even deadlier.

Thus, there is an urgent medical need for new drugs and treatment regimens that can better manage the latent tuberculosis infection [7,8,9]. Although over a dozen anti-tuberculosis drugs are available, a treatment regimen of significantly less than six months has not been fully established. For tuberculosis chemotherapy, there currently exist four first-line drugs, six second-line drugs, four approved drugs with anti-TB activity, and at least four promising drugs in clinical trials [10]. These drugs target many aspects of *M. tuberculosis* cellular structures and biological processes, e.g. transcription, protein synthesis, cell wall synthesis, catalase-peroxidase enzyme, ATP synthesis, DNA replication, and cofactor synthesis. Although shorter (<6 months) treatment regimens were created with a combination of the existing drugs, the relapse rates of the shorter regimens were consistently higher than that of the standard treatment regimen [11,12].

The standard 9-month treatment regimen was based on 50-year clinical practices but the exact mechanism of how it works remains unclear. It is well-known that isoniazid is only active against growing *M. tuberculosis* [13] and is inactive against anaerobic bacteria [14]. The traditional view is that persistent *M. tuberculosis* resides within hypoxic granuloma lesions in a static mode. This however does not fully agree with the fact that isoniazid is 90% effective in eliminating persistent *M. tuberculosis* from latent tuberculosis infection patients. On the other hand, if *M. tuberculosis* resides in aerobic microenvironments in latent tuberculosis infection, the treatment would not have taken as long as 9 months to kill the bacilli. There has been an on-going debate over the microenvironments in latent tuberculosis infection where *M. tuberculosis* resides [3,15,16,17]. The complex physical, biochemical, and microbiological milieu of *M. tuberculosis* in tuberculosis disease have been a major obstacle that hinders the development of shorter treatment regimens to eradicate the disease.

Even after years of pursuit to understand the biology of this pathogen, we have only been able to uncover a very small percentage of its *modus operandi*, especially in dormant cells. At one point of time when tuberculosis was ravaging the world population, effective chemotherapy was believed to be successful in halting the spread of tuberculosis. However, the disease returned many years later in the form of reactivated bacilli from the latent state. It was realized that chemotherapy does not always completely abolish the bacteria from the host [18,19], but rather the bacteria undergoes a transformation due to environmental stress which allows it to change its metabolic activity and transition into dormant or persistent cells – a phase which came to be known as latent tuberculosis [5,20,21,22]. 

It was soon realized that very little was actually known about the biology of *M. tuberculosis* and that the bacilli was a complex organism in the host. The complexity of *M. tuberculosis* arises from the fact that it is able to survive and proliferate inside macrophage phagosomes in spite of exposure to various stresses in the phagosome, modulate the host phagosomal environment [23], acquire nutrients required for growth [24,25] and finally change its metabolic state when the macrophage is able to halt its proliferation. Efforts were soon underway to study the biology of actively replicating bacilli as well as the dormant forms. The sequencing of *M. tuberculosis* genome was an important step forward in understanding the bacteria [26]. Biochemical studies coupled with transcriptome analysis in *in vitro* and *in vivo* analyses started to unravel the genes expressed during the adaptation of *M. tuberculosis* to different stresses in the phagosome and genes expressed during an infection [27,28].

Another important aspect in the pathobiology of tuberculosis was to understand the immune system. Any pathogen needs to successfully overcome the non-specific and specific immune reactions in order to establish an infection. Hence, understanding macrophage biology also becomes important in conjunction with the biology of *M. tuberculosis*. 

With advancing technologies, the trend towards systems biology emerged strong. Along with transcriptome analyses, proteomic and metabolic profiling also began to gain ground. With advances in studying the proteome from the conventional 2D gel analysis to high sensitivity analyses using mass spectrometers, studying the proteome is seen as a promising candidate for systems biology approach. The fundamental advantages that proteome analyses offered over transcriptome analyses was that it focused on functionally relevant species and is important for drug related information since drugs primarily target proteins. However, the field of proteomics is still evolving. Whereas transcriptome analyses can identify thousands of genes differentially expressed in an organism, proteomics is still struggling to get to the depth of analysis that a transcriptome study does [29,30].

Classical proteomics has concentrated on determining abundances of various proteins in the proteome [31,32]. Quantitating differentially expressed proteins has given useful information regarding the status of the cell at a given point [30,33]. At the global level, proteomic analyses do not permit the reliable quantification of absolute abundances. Hence, most proteomic studies concentrate on relative abundances of proteins in two separate states. However, any pathogen does not have definite steady states in the host and is constantly trying to overcome host immune responses in order to survive and establish infection. Thus, interplay between the host and the pathogen creates a dynamic response in the pathogen and the host which is not explored using classical proteomics. The dynamic nature of the pathogen is one of the primary reasons why such a discrepancy exists between the available transcriptome and proteome data [34].

In this review, we discuss the knowledge obtained on the biology of *M. tuberculosis* from current proteomic studies. Additionally, we explore strategies to study the dynamics involved in a proteome of *M. tuberculosis* using the protein turnover analysis technique. Protein turnover along with relative protein abundance values can help analyze the dynamics associated with a proteome system. Using our analysis of protein turnover and abundance values in stressed *M. tuberculosis* as an example, we show that protein turnover measurements are more sensitive than relative abundance measurements and that a combination of both measurements combined with transcriptomics can depict a more complete picture of the pathogen in the host. The analysis of dynamic responses of the pathogen might help in new drug discovery. More importantly it might help in understanding the biology of persistent *M. tuberculosis* on which information is still very limited.

## 2. Proteome Analyses in Mycobacteria

Mycobacterial proteomes have been analyzed over the years in a number of different conditions involving nutritional, acidic, oxidative and low oxygen stresses. Here we review some of the important findings in mycobacterial biology upon examination of the response of the bacilli to various stresses.

### 2.1. Modulation of host phagosomal environment

The initial stage when a bacterium enters the macrophages is the focal point of whether the bacillus is successful in establishing an infection. *M. tuberculosis* is communicated through the air passage. It establishes infection primarily in the lungs. The first resistance that it encounters is from alveolar macrophages. These macrophages engulf the bacteria through phagocytosis for pathogen killing, infection neutralization and antigen presentation. One of the important things that was learnt from early studies on *M. tuberculosis* is that *M. tuberculosis* is an intracellular parasite known to reside inside macrophages and that it can modulate the host cells in order to survive and proliferate. Many studies have focused on characterization of modulated phagosomes as well as the mechanisms of modulation by the bacilli. These studies have shown that *M. tuberculosis* was able to live in a niche or microenvironment which is created inside the phagosome [25]. Two aspects became important from this knowledge. The first was to understand how the phagosome responds to intracellular pathogens and the second was to decipher how the bacilli were able to overcome the stresses mediated by professional phagocytes.

Macrophages internalize various particulate materials through the concerted action of several surface receptors. The engulfment leads to the formation of membrane bound organelles i.e. phagosomes. Phagosomes are dynamic bodies. Each phagosome recruits a variety of proteins through endosomal pathways. When it finally recruits acidic vesicles called lysosomes, it matures into phagolysosomes. Proteome analyses have revealed many interesting characteristics about the nature of phagosomes and phagolysosomes. Phagosomes contain many GTPases and hydrolases and proteins are recruited by the phagosomes as they mature along the endosomal pathway. Many proteins are recycled and hence proteins found in the initial phagosomes may not be found in the final composition of the phagosome. For example while hydrolases such as cathepsin A and β-hexosaminidase are already present in high amounts in the early phagosome, other hydrolases such as cathepsin S and cleaved form of cathepsin D appear later during phagolysosome maturation [35]. Also, many phosphoproteins such as Rab are recruited by the phagosome indicating that phagosome maturation and action is a result of signaling processes involving a cascade of phosphoproteins [36].

Another important point to be noted is that the composition of the phagosome varies with the type of material in it. Comparison of phagosomes containing intracellular pathogenic *versus* the non-pathogenic mycobacterial species have indicated that the phagosomal composition is dependent on the pathogenic species in the phagosome [25]. Recruitment of proteins such as NRAMP1 and NRAMP2 seems to be important for the regulation of different inorganic ions in the phagosome. Phagosomes containing intracellular pathogens have differing levels of inorganic ions. Inorganic ions such as iron, manganese and zinc are critical for the growth and survival of mycobacteria and secretion of biomolecules to alter the composition of phagosomes is known to occur with many pathogens such as in *Salmonella* species [37,38].

In *M. tuberculosis*, studies have shown that the bacilli secrete siderophores to chelate iron away from the host storage molecules such as transferrin and lactoferrin [24,39]. Apart from acquiring important inorganic ions, it is also important for the bacilli to stop the maturation of the phagosome into an acidic compartment. The phagosome matures into an acidic vesicle which then recruits hydrolytic enzymes to help neutralize the pathogen to proceed for antigen presentation [23,40].

Analysis of intraphagosomal *M. tuberculosis* proteome comparing proteins from a broth grown *versus* intraphagosomal bacilli using 2D gel analysis revealed proteins such as phosphoglycerate mutase I, lipid carrier protein (Rv1627c), TrkA, a putative potassium uptake protein and other conserved hypothetical proteins. Apart from this, other proteins involved in a global stress response such as peptidyl-prolyl isomerase (Ppi) was found to be involved in both low pH conditions and low iron conditions. Increase of putative lipid transfer protein Rv1627c reveals that mycobacterial lipids may play an important role in the virulence of *M. tuberculosis* [41]. Presence of these proteins indicates that various proteins are transcribed and secreted in the phagosome to facilitate the survival of the bacilli in the host macrophages. 

Alteration of phagosomal composition is also affected by the intervention of various signaling processes in the macrophage leading to arrest of maturation of the phagosome. As stated before, phagosomal maturation depends on several signaling cascades. Interference with host signaling achieves the sole purpose of survival for *M. tuberculosis* by inhibiting phagosomal maturation and phago-lysosomal fusion. Through heterogeneous secretion of proteins, lipids and glycolipids in the phagosome, the bacilli are able to interfere with phagosomal maturation, antigen presentation, apoptosis and stimulation of bactericidal responses triggered by host signaling cascades. Glycolipid lipoarabinomanan (LAM) in the bacilli gets modified into Man-LAM by the addition of mannose and is an abundant glycolipid in pathogenic mycobacteria. Interference with host signaling pathways by mycobacteria is also accomplished by altering levels of secondary messengers such as Ca^+2^ and by interfering with phosphoinositide 3-kinase (PI3K) signaling. Inhibition of lipid kinases which are important for phagosomal maturation is another aspect of interference with the host signaling. Macrophages, when infected with potentially harmful pathogens have the ability to undergo apoptosis which halts the spread of harmful pathogens. Host macrophages infected with *M. tuberculosis* have altered apoptotic pathways due to phosphorylation of the apoptotic protein Bad which does not allow other apoptotic proteins downstream to be activated. *M. tuberculosis* also inhibits the immune system by inducing the production of IL-10. It is also known to modulate the activation of MAPK and JAK-STAT pathways which induce pro-inflammatory responses thereby successfully downregulating the host immune response against an infection [42]. Interference with host signaling pathways is not just a single method that *M. tuberculosis* uses to arrest phagosomal maturation or phago-lysosome fusion. Walburger *et al*. [10] through mutational analysis showed that *M. tuberculosis* secretes protein kinase G into the macrophage. It was one of the first instances where it was shown that a eukaryotic-like signaling pathway was used to modulate the host phagosomal composition thereby promoting survival of the bacilli.

The conclusion from the various responses of *M. tuberculosis* listed above indicates that the macrophage phagosome and the bacilli display dynamic responses within the system. By appropriating various nutrients needed for survival in the phagosome and at the same time facilitating its survival by secreting biomolecules into its environment, *M. tuberculosis* achieves the balance required to survive, proliferate and establish an infection.

### 2.2. Mycobacterial response to low iron stress

Out of all the micronutrients that *M. tuberculosis* needs to grow and survive, iron is probably the most important one. Many studies have proven that an increase in levels of iron in the host exacerbates the onset of tuberculosis [43]. Even though iron is an abundant metal, it is not readily available in the free form. It is then of no wonder that the bacilli spend significant cellular resources in acquiring this metal. It is not an easy task since in the event of an infection, the host immune system sequesters iron away from the pathogen as a nonspecific immune response [44]. Macrophages activated by IFN-γ tend to lower the concentration of iron binding molecules especially transferrins in the macrophage endosomes. Iron acts as an important co-factor for as many as forty different enzymes, the most significant of them being the respiratory enzymes [45]. Apart from this, iron is also needed for protective enzymes such as catalase peroxidase KatG and super-oxide dismutase (SOD) that scavenges free radicals [46]. Mycobacteria are more sensitive to hydrogen peroxide mediated stress in the absence of iron [47]. In order to acquire iron in the host, *M. tuberculosis* secretes siderophores which chelate away iron from the host transferrin and lactoferrin [39,48].

Siderophores are typically salicylate-containing biomolecules. In *M. tuberculosis*, these siderophores are called mycobactins. *M. tuberculosis* contains two types of mycobactins, one is a soluble form and the other is membrane bound form. It is known that both mycobactins act in a concerted manner to transport iron into the mycobacterial cell. However, the exact mechanism by which both act is not yet completely deciphered [49]. Since drugs designed to either block siderophore action or mimic siderophores can be used against the bacilli, mechanism of mycobacterial siderophore action is currently amongst the leading topics of research [50,51]. Although iron is an important metal, its uptake and use has to be tightly regulated because of its ability to form free radicals in the presence of hydrogen peroxide (Fenton’s reaction) [52]:

Fe^+2^ + H_2_O_2_ → Fe^+3^ + OH^•^ + OH^-^

The regulation of iron metabolism is governed by the iron dependent regulatory protein (IdeR) which controls the uptake of iron by mediating the genes responsible for the synthesis of siderophores. The level of iron in the cell is important for both the host and the pathogen. Iron is not only required for immune regulatory functions, it is also needed for the bacterial multiplication and survival. More interestingly, iron has been known to enhance the action of isoniazid (INH) and pyrazinamide on *M. tuberculosis* [53,54]. Hence iron is an important virulence factor. Response of *M. tuberculosis* to low iron levels have been recorded at the mRNA and protein levels. Most of the global response due to iron starvation is by rearrangement in the synthesis of various metabolic enzymes [55]. The bacilli seem to regulate its metabolism to counter the stress imposed by low levels of iron [21]. Mycobactin producing genes are also upregulated in response to low iron level. Proteins such as bacterioferritin (Bfr), which is involved in the storage of iron seems to be downregulated when iron level is low. It is interesting to note that proteins such as KatG, SOD are downregulated in response to a low iron condition [47,56]. This serves the purpose of not spending energy for the synthesis of proteins not needed in the absence of iron. The downregulation of KatG under low iron conditions is interesting because KatG is the primary catalase peroxidase and peroxynitritase in *M. tuberculosis* [46]. The downregulation of such an important protein might leave the bacilli vulnerable to an oxidative stress that is part of immune response to the pathogen infection. Studies involving strains that have a non-functional KatG have shown that other proteins such as alkyl hydroxy-peroxidase C (AhpC), thiredoxin C (TrxC) and thiol peroxidase (Tpx) seem to take over the function of protecting the cell from oxidative damage when KatG is unavailable [57,58,59,60,61]. The situation when KatG is unavailable is not unimaginable because most INH resistant strains have an inactive KatG in a mutated and hence non-functional form [62]. Another important iron regulator protein is the ferric uptake regulator (Fur). Fur is upregulated under a high iron condition and is also known to control the transcription of genes involved in bacterial iron uptake. Analysis involving both Fur and IdeR showed that both regulators are important for the maintaining and checking the levels of iron in the bacterial cell. Elaborate reviews explaining the nature of regulation of Fur and IdeR can be found in [49,63,64] Low availability of iron is also known to induce dormancy response as a response to starvation or lack of nutrients [21].

### 2.3. Acidification of phagosome

As explained earlier, acidification of phagosomes is an important phenomenon in the macrophage as it leads to neutralization of the pathogen since most enzymes in the bacteria are rendered inactive under acidic conditions. Most *in vitro* studies on the response of *M. tuberculosis* to acidic pH have been carried out with transcriptome analysis [28,65]. Two studies analyzed the transcriptome of *M. tuberculosis* and *M. smegmatis* at acidic conditions (pH 5.0 and pH 5.5) and identified 291 and 81 differentially expressed genes respectively. Out of these differentially expressed proteins, the most significant were lipF and SigF genes which were induced >1.5 fold. These two genes were not induced in the bacilli in J774 macrophages infected with live *M. tuberculosis* which correlates with the data that phagosomes containing live bacilli fail to acidify [28,66,67]. A study conducted on phagosome *M. avium* revealed even though LAMP-1 was acquired by the phagosome, it failed to acquire vesicular proton-ATPase which is needed for acidification and may be an important step in inhibiting the acidification of the phagosome by mycobacteria [68]. 

*In vitro* low pH proteome analysis studied in our lab using non-pathogenic *M. smegmatis* strain identified *ca.* 1,070 proteins totally, out of which 241 proteins were differentially expressed. Gene ontology (GO) studies showed that three GO terms were upregulated involved stress response (GO:0006950), amino acid metabolic processes (GO:0006520) and monosaccharide metabolic process (GO:0005996). GO terms that dominated downregulated proteins were transmembrane ion transport (GO:0034220), polyol metabolic process (GO:0019751), nucleotide-sugar biosynthetic process (GO:0009226) and polysaccharide metabolism (GO:0005976). Repression of ion channels can be seen as an adaptation in response to higher proton concentration in the outside environment of the mycobacterial cell. Other responses seem to indicate that metabolic systems are regulated in the bacilli when exposed to low pH especially sugar metabolism with increased monosaccharide metabolism compared to polysaccharide metabolism as obtained by KEGG pathway analysis [69].

### 2.4. Exposure to nitric oxide and dormancy

Apart from nutrient starvation and acid stress, *M. tuberculosis* is also exposed to oxidative stress in the form of reactive nitric oxide molecules (NO). NO is generated by the iNOS pathway that metabolizes L-arginine. NO is an uncharged molecule composed of seven electrons from nitrogen and eight electrons from oxygen. This combination of the 15 electrons results in the presence of an unpaired electron that makes NO paramagnetic and a radical. The majority of biological molecules contain bonds filled with two electrons and are not reactive toward NO. However, a select range of molecules that have unpaired electrons in their outer orbital will be highly susceptible to a reaction with NO, such as a haem iron. Because iron-containing enzymes and proteins are abundant in a cell, NO can potentially bind to these biomolecules as a free radical and render them unstable [58]. Because NO is a potent vasodilator, the synthesis of NO takes place for a very short time in the macrophages. Transcriptome analyses have shown that an exposure to NO induces dormancy in the bacilli. Short exposure to NO resulted in the differential expression of a specific set of genes called the DosR/S regulon or the dormancy regulon [70]. The genes under the regulation of DosR/S are called the DosR regulon, which consists of *ca.* 53 genes. DosR is important to initiate the shift from an actively growing state to a non-replicating state or dormancy. The effects of DosR were shown to be somewhat transitory and to lead to the definition of an ‘enduring hypoxic response’ (EHR) [71]. There are a set of 230 EHR genes induced after the transitory response of the DosR regulon. These genes are independent of the DosR/S-mediated initial response. They are induced later after the initial DosR response, and are sustained during the dormancy phase to suggest that they could be more relevant to the dormant state of *M. tuberculosis*. Because many tuberculosis patients might receive treatment long after the initial infection, the EHR genes should represent a better pool of drug-target candidates against dormant *M. tuberculosis* [72]. 

Dormancy is an important aspect in mycobacterial pathogenesis since it leads to a population of bacteria in the host known as latent bacteria. The hallmark of latency is that the bacilli reside in a specialized compartment of dead cells and necrotic tissue known as a granuloma where hypoxic and low nutrient conditions occur. To adapt, the bacilli lowers its metabolism and can reactivate later if the host immune system is compromised [20,22]. For the past many years, significant efforts have been put into not only understanding the biology behind dormant cells but also different strategies to eliminate the dormant cells using conventional drugs [73,74]. One of the important characteristics of dormant cells is their low level of metabolism along with their non-replicating status. A proteome analysis of non-replicating persistent *M. tuberculosis* in the NRP-1 and NRP-2 stages of the Wayne’s model of dormant cells revealed that the global protein expression decreased under both NRP-1 and NRP-2 conditions. A total of 38 and 128 proteins were found to be differentially expressed more than 2-fold in the NRP-1 and NRP-2 stage respectively. Of the downregulated proteins in the proteome most of them corresponded to proteins involved in transcriptional and translational machinery which may be required to adapt to low oxygen levels. Of the upregulated proteins, many proteins that were detected had unknown functions. Trehalose metabolism proteins were upregulated, which coincides with the fact that trehalose has been known to be a stress protectant and also serve as a carbon source [75]. Starck *et al*. [76] showed that *M. tuberculosis* cells grown under anaerobic conditions had α-crystallin homologue and GroEL2 along with few metabolic proteins that were found to be upregulated under starvation in other independent studies [27,77]. The above results show that hypoxic stress and an exposure to NO enforce some similar kind of stress response patterns in proteins related to carbon metabolism and stress response proteins. 

In macrophages, initial progress of mycobacteria can be halted by the host immune system or by the combination of an anti-TB drug that targets actively replicating bacteria. The host immune system helps to initiate a set of stresses such as deprivation of iron, low oxygen, and exposure to NO. The exposure of the bacilli to those stress factors or drugs, however, might also initiate the dormancy response of the bacilli that in turn renders a subpopulation of the bacilli resistant to the host assault or drug treatment. At this stage, the tuberculosis infection might appear to be cured but could also resurface later due to a weak immune system resulting from aging, poor nutrition, or co-infection by HIV [15,78]. Because dormancy is related to starvation and is an important aspect of mycobacterial pathogenesis, most proteome studies are directed towards understanding what drives the bacilli towards dormancy and how does the bacilli cope with survival at the dormant state.

## 3. Turnover with Respect to Proteome

Protein turnover is an important biological phenomenon in an organism and shares an equally important role with gene transcription and protein translation [79]. Synthesis of new proteins and degradation of old ones form a dynamic process in an organism. Turnover does not only help to clear old proteins but also aid in a fast adaptation to a new condition or environment by altering their protein turnover rates [80]. Apart from this, turnover also brings into equation the action of new proteins without much strain on the resources of an organism. Previous studies done on turnover and their rates were concentrated on single proteins [81]. Some early global protein turnover studies involved identification of different *E. coli* proteins that might have different turnover rates. One of those earlier studies showed that a dynamic state for individual proteins existed in non-growing as well as growing cells [82]. Studies of turnover tend to give a dynamic view of the changes occurring in the abundance of the protein. When being applied to a larger scale of the proteome, it allows us to study the dynamic nature of the entire proteome [79].

The *M. tuberculosis* genome contains approximately 4,000 protein coding genes and most dormancy studies involving the proteome detected far fewer proteins that were differentially expressed. Out of those proteins that were detected, there were mostly metabolic proteins and few stress response proteins [75]. It can be explained that for studying non-replicating persistent *M. tuberculosis*, current technology might have limitations to detect more subtle responses. 

It is widely known that in very few cases a very strong correlation is found between transcriptome studies and proteomic investigations. Most proteomic studies are able to successfully identify hundreds of proteins in *M. tuberculosis* through the use of sensitive mass spectrometers available today. However, each study finds extremely less number of proteins differentially regulated especially in the case of intraphagosomal bacteria or persistent bacteria. In a proteomic analysis of intraphagosomal *M. tuberculosis* Mattow *et al*. [41] have acknowledged the fact that there appears to be very little correlation between their previous transcriptome studies and current proteomic analysis. 

One of the caveats of studying the proteome is that it focuses on functionally relevant species. Therefore, an insight into the proteome provides more direct interpretation of a drug activity. With advances in mass spectrometry an investigation of complex proteomes has become more accessible. This upward trend however has mostly been in the field of instrumentation [31]. Proteomics has so far focused on steady state abundances of various proteins [83] to compare increase or decrease of protein abundance in one state *versus* another. Even though this approach brings in a lot of information, it does not completely cover the flux in the system that arises due to the dynamic nature of the organism e.g., the dynamics associated with *M. tuberculosis*, when it tries to constantly overcome the stresses in the phagosome. One advantage of proteome studies over transcriptome analyses is that transcriptomics cannot study the dynamics associated with a system. Of course many time course analyses have been carried out but the question still remains as to how much of that data is functionally relevant to gene products that carry out the functions. The point is that one needs a technique which not only utilizes the data available from transcriptome studies but also to focus on the proteome so that the technique will take into account the dynamics associated with the system. With the recent advances in high-resolution mass spectrometry, a protein turnover analysis at the global level promises to bring about valuable insights into the dynamic nature of a proteome [79]. Studies of global protein turnover in an organism investigate two main aspects that contribute to a steady state protein abundance in an organism i.e., protein synthesis and protein degradation. In logarithmically growing bacterial cells, the rate of protein synthesis is much greater than that of protein degradation. Under stresses that lead to a non-replicating state, the rate of protein degradation increases dramatically relative to that of protein synthesis leading faster protein turnover. Under these conditions, the coupling of gene transcription and protein translation will not be the only factor that determines the protein abundance. Thus, the discrepancy arises between transcriptomics and proteomics data. The dynamic process of protein turnover determines the abundance of the protein in the cell [55,56,84,85]. In addition, protein secretion also affects the steady state abundance of a protein in the cell [56]. Hence, to fully comprehend the abundance of proteins in the cell, methods have to be developed that not only takes into account gene transcription, but also protein synthesis, degradation, secretion, and probably modification as well.

### 3.1. Protein turnover discerns more subtle changes in a cell

The advent of highly automated high-resolution mass spectrometry technology promises to bring about in-depth insight into the dynamic nature of a proteome at the global level. The work done by Pratt *et al*. [85] demonstrated the determination of protein degradation rate constants in a steady state population of yeast grown in a chemostat. The authors used isotope labeling along with 2D gel analysis to study protein turnover and advocated that protein turnover is ‘a missing dimension in proteomics.’ Another study done by Cargile *et al*. [84] labeled *E. coli* cells with ^13^C to study the relative synthesis over degradation ratio (S/D). These pilot works demonstrated the global analysis of protein turnover with individual protein identifications but their data were not correlated with abundance values.

In our laboratory, we used *M. smegmatis*, a non-pathogenic surrogate of *M. tuberculosis*, to study the protein turnover under the stresses of low pH and low iron. As mentioned before, *M. tuberculosis* is exposed to both stresses in the phagosome until the bacilli overcomes them. We were interested in studying the effect of low pH and low iron on the global protein turnover [55]. The cells were grown with two different methodologies for both types of stresses. For the pH stress the cells were initially grown in ^14^N containing media at pH 7.0. Once the cells were in the initial log phase, the cells were divided into two flasks and the media was doped with 50% ^15^N and the pH was reduced to 5.0 in one of the flasks. The cells were harvested after one doubling and analyzed for protein turnover using LC/LTQ-FTMS. For low iron analysis, the cells were first grown in ^15^N containing media until mid-log phase. The cells were then collected by centrifugation and the media was then exchanged with ^14^N containing media. The cells were then allowed to grow to one doubling and harvested to be analyzed by LC/LTQ-FTMS.

Turnover analysis of *M. smegmatis* under both stressful conditions revealed two different patterns. In the low pH condition, many proteins had increased turnover at pH 5.0 as compared to pH 7.0. It was an obvious reaction because the bacterium has to readjust its proteome in order to counter the stress posed by increased proton concentrations. The correlation coefficient for the low pH shock cells was small which indicated that the proteins in the cells exposed to pH 5.0 underwent extensive readjustment in different directions. In the low iron stress the correlation coefficient being high suggested that either there was not much rearrangement of turnover values or most proteins had changes in a similar direction. Proteins like KatG and Tpx which are important for protection of mycobacterial cells against oxidative stress had low protein turnover values in both low iron as well as low pH conditions. A recent study on *M. tuberculosis* Tpx suggested that it may be an important protein against oxidative stress since Tpx mutants were unable to survive in the macrophages in an infected mouse model. However, it would be interesting to analyze how the low turnover of Tpx correlates with the survival of mycobacteria in the cell. One protein that was found to have increased synthesis was RNasE (Rne). It is known that under certain stress conditions, cells downregulate their metabolism until suitable growth conditions can be found again. RNaseE, an important enzyme involved in the turnover of mRNA, is a key for bacterial mRNA degradation and processing. Low iron conditions may trigger a necessity to increase degradation of mRNAs in the cell in order to inhibit protein synthesis. This may necessitate an increased synthesis of RNaseE as indicated by the increase in synthesis over degradation ratio. Increased synthesis of RNaseE may be important for a tighter regulation of mRNAs and for survival in adverse environmental conditions. RNaseE levels were also found to increase during adaptation to starvation. We suspect that an increase in the synthesis of RNaseE may be important to dormant cells as well.

**Figure 1:**
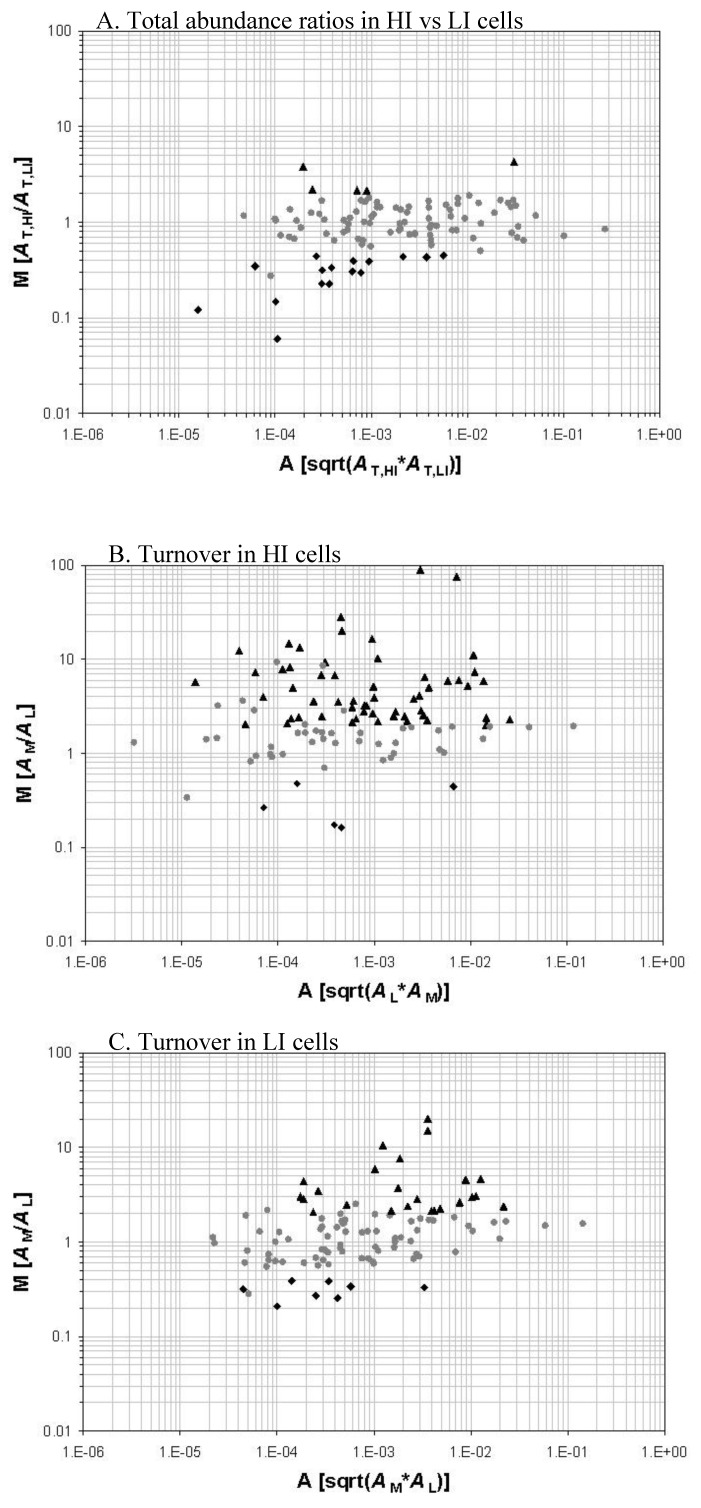
Protein relative abundance and turnover of iron-starved *M. tuberculosis* cells in response to a high-iron (HI) and low-iron (LI) condition. The abundance of a protein is represented by an extracted ion-chromatographic intensity (*A*). A protein is quantified for its old fraction abundance (*A*_L_), new fraction abundance (*A*_M_), and total abundance (*A*_T_). *A*_T_ is the sum of *A*_L_ and *A*_M_. *A*_M_/*A*_L_ represents the S/D *i.e.,* protein turnover. The three M-A plot panels illustrate the total abundance ratios of protein between the HI and LI cells (Panel A), the protein turnover in the HI cells (Panel B), and the protein turnover in the LI cells (Panel C) respectively. Those proteins with a >2-fold change (*p <* 0.05) in total abundance ratio or turnover are marked with black triangles and diamonds. Adapted from reference [56] with permission.

We demonstrated the successful study of protein turnover at the global level under different stress conditions. There is another open question, however, that how the protein turnover values correlate with protein abundances. To compare protein abundance and protein turnover values in *M. tuberculosis* under iron-regulated conditions [56], we collaborated with Dr. Issar Smith’s laboratory at The Public Health Research Institute (TPHRI) to grow the cells in an iron replete and iron depleted condition. *M. tuberculosis* initially grown in iron depleted unlabeled media containing a ^14^N nitrogen source. Log-phase cells were transferred to two different flasks containing ^15^N-labeled media. One of the flasks contained iron whereas the other was still iron depleted. In summary, a comparison was made between cells that were transferred from a low iron to a high iron condition. The cells were harvested and analyzed for protein turnover using the high resolution nano-LC/LTQ-FTMS system (Figure 1). The proteins de novo synthesized in the ^15^N-labeled media are the new fraction. The proteins remain unlabeled are the old fraction synthesized prior to the transfer to the ^15^N-labeled media and survive the degradation and secretion processes.

The data obtained from the comparison of protein abundance and protein turnover values showed that protein turnover is a much more sensitive measurement to discern the changes in the proteome than an abundance measurement alone. This improved ability of turnover analysis to discern more subtle changes in protein synthesis, degradation, and secretion activities is due to the fact that newly synthesized (labeled) and pre-existing proteins (unlabeled) are quantified separately so that the level of de nova protein synthesis, protein degradation, and protein secretion can be deduced separately.

Upon the transfer of late-log phase cells from a low iron to a high iron media, protein abundance measurements showed that out of the 104 proteins that we identified, only five proteins were upregulated and 16 proteins were downregulated in the HI media. Relative abundance of KatG was upregulated in cells grown in the high iron media. 

Protein turnover analysis of the proteins compared between cells grown in a low iron and a high iron media showed that more proteins had increased synthetic activity in the high iron grown cells. The S/D had increased for 24 proteins in the cells grown in the low iron media. Eight proteins had decreased turnover. However, for cells grown in the high iron media, 56 proteins had increased S/D and five proteins had decreased S/D. Comparison of protein abundance measurements to protein turnover measurements clearly suggests that protein turnover does give more information to uncover the dynamic response of the proteome (Figure 1).

In addition to providing the information about synthesis and degradation of proteins, the protein turnover analysis can also provide information regarding whether a protein has been secreted when the protein turnover values are analyzed together with the protein abundance measurements. In our study of proteome dynamics, we found that some proteins had low changes in relative abundances even though their synthesis had increased significantly. As stated before the relative abundance of a protein in the cell can be affected not only by synthesis or degradation but also by a secretion process. In our turnover analysis, we found discrepancies between protein abundance values of certain proteins and their turnover values. A previous proteomic study of *M. tuberculosis* culture filtrates showed many of those proteins to be secreted into the culture filtrate. Proteins such as FbpC2, KatG, and the mammalian cell entrance protein Rv0172 were also predicted to be secreted [86].

These results support that protein turnover in combination with abundance analysis could predict the secretion of proteins and reveal the interconnected roles of protein synthesis, degradation, and secretion in determining the protein abundances in cells. These analyses illustrate that protein turnover can divulge information that classical proteomics does not provide. The integration of data from transcriptome studies, abundance measurements and turnover analyses will likely provide a more complete picture of the dynamics associated with the proteome. To some extent, it will probably reconcile the discordances between transcriptome and proteome analyses.

### 3.2. Implication of proteome turnover studies in mycobacteria

*M. tuberculosis* is a potent human pathogen parasitizing macrophages. It induces vigorous immune responses, yet persists inside macrophages, evading host immunity. Although a variety of control and eradication measures have been implemented against tuberculosis such as vaccination, aggressive chemotherapy, and public health surveillance, tuberculosis still remains a major global health problem to continue to cause nearly two million deaths and nine million new infection cases per year. *Ca*. one third of the world population is infected with latent *M. tuberculosis*.

Efforts to search for effective vaccines against tuberculosis and new drugs that can rapidly sterilize latent *M. tuberculosis* are hindered by the lack of information about the metabolism and immunogenicity of the intracellular bacterium in a latent form. There are two important questions related to the chemotherapeutic agent and vaccine development efforts: a) does intracellular *M. tuberculosis* synthesize unique proteins immunologically important? and b) is the protein synthesis profile of latent intracellular *M. tuberculosis* so different from that of *in vitro* models that new drug target candidates are needed for faster treatment of latent *M. tuberculosis*? Elucidation of the *M. tuberculosis* protein synthesis within infected macrophages will provide valuable information to further understand the molecular basis of tuberculosis latency, to provide novel targets for drug development, and to possibly discover a more potent vaccine candidate.

With the advent of high-precision and automated mass spectrometry instrumentation to support large-scale proteomic studies, protein turnover analysis at the global level potentially has an increasing importance for biomedical research [79]. One application of protein turnover analysis would be to study the *M. tuberculosis* proteome dynamics at its non-replicating state in an intracellular environment or under an *in vitro* culturing condition. Whereas over a hundred research articles have been published on mycobacterial proteomes, only a few dealt with non-replicating *M. tuberculosis* [27,75,87]. Information about protein turnover in non-replicating or dormant *M. tuberculosis* is scarce in the literature. With the potential importance of proteome dynamics in bacterial cell sporulation or dormancy [81,88,89,90], a study of mycobacterial protein turnover at the global level [55,56] will likely help to advance our understanding of the molecular basis of *M. tuberculosis* persistence. 

The metabolic requirement of *M. tuberculosis* in latency remains unclear and difficult to study. The long therapeutic regime required to treat latent tuberculosis infection is probably in part due to the lack of a direct target that is specific to dormant *M. tuberculosis*. While there are new drug treatment regimes and several new anti-tuberculosis drugs in the development pipeline that aim to shorten the treatment period and to overcome multi-drug resistant strains [72], most of these new drugs are still based on existing classes of antimicrobial compounds that target the conventional pathways and molecular machinery that are critical for the growth of *M. tuberculosis*. These drugs could still be countered by drug resistant strains that emerge from the non-adherence of a prolonged regime against latent tuberculosis infection. Thus, the need to discover novel drug targets, especially those against dormant bacteria, is urgent.

A ‘simple but nonetheless vexing problem’ [72] in target discovery against non-replicating *M. tuberculosis* is that many methods rely on a growth-inhibition measurement to assess the effect of drug treatment. We showed that, at a global level, a protein-turnover measurement was more capable to discern protein synthesis and degradation activities than a protein relative-abundance measurement alone [56]; those data suggest that protein dynamics analysis with concomitant turnover and abundance measurements could potentially add a valuable alternative to the drug target discovery problem for non-replicating *M. tuberculosis*. The concomitant turnover and abundance measurement approach could also be useful to detect and validate drug treatment effects at an early phase during which the most relevant drug effect can be isolated from other non-specific cell stress responses.

We anticipate that protein turnover analysis will also be a useful tool to profile the proteins uniquely expressed in intracellular *M. tuberculosis i.e.,* the bacilli grown in macrophages. In one conceivable experimental design, for example, one could first label culture broth-grown *M. tuberculosis* cells with a stable isotope, such as ^15^N or ^13^C, and then use the labeled *M. tuberculosis* cells to infect macrophages grown in an unlabeled culture medium. In this way, new proteins synthesized after the *M. tuberculosis* cells enter the macrophages will be unlabeled and will be distinguishable from the old proteins pre-existing in the labeled *M. tuberculosis* before infection. Because the abundance of the old and new proteins could be determined separately [56], the proteins more prone to degradation could also be profiled. Based on the quantitation of old, new and total protein abundances, one can deduce the proteins that are probably secreted into a phagosomal compartment. The secreted proteins, especially in an intracellular environment, are more likely to involve in the modulation of immune response and antigen interaction. Those proteins secreted in an intracellular environment would be more likely to be an effective vaccine candidate.

## 4. Conclusions

Protein turnover is an essential part of a metabolically active organism. It is also one of the important parameter affected when the organism is adapting to an environment. Study of protein turnover at the global level helps us to understand the dynamics of the proteome as it changes from one state to another. Proteome dynamics involves a continuous degradation and synthesis of proteins. The steady state abundance of a protein in the cell is a function of its synthesis and degradation. *M. tuberculosis* undergoes significant changes at the proteome level as the bacilli tries to establish an infection while residing inside the macrophages. This correlates to changes in the synthetic and degradative processes in the mycobacterial cell. Since an increase in protein abundance can be the result of an increase in synthesis or decrease in degradation of that protein or both, similarly, a decrease of protein abundance can be a result of either decreased synthesis or increased degradation or both. A change in both the synthetic and degradative processes in the same direction may not affect the net abundance of the protein to a large extent; however, a change in turnover should be detectable. Additional importance of protein turnover studies comes from the fact that protein abundance and transcriptome analyses can augment the information of turnover data thereby deriving information on proteins that are differentially expressed and also proteins which are secreted outside the cell. Until now, proteome analyses did not give us that kind of information.

*M. tuberculosis* is able to overcome a weakened immune system through a variety of stress response mechanisms described above and proliferate. However, in an immuno-competent host, the bacilli might proliferate for some time before its growth is arrested. This leads to another phase of mycobacterial physiology i.e. the dormant or latent phase characterized by non-dividing cells and a low metabolic profile. The biology of dormant non-dividing state of mycobacteria is still a mystery. Current analytical methods involving conventional proteomic approaches are being found inadequate at deciphering the biology of dormant bacilli.

With recent advances in quantitative proteomics and mass spectrometry, it is now possible to study global protein turnover at many different phases of an organism. The data obtained in our turnover studies were from growing populations of *M. tuberculosis*. However, the sensitivity of our analyses suggests that one could extend the turnover analysis to dormant *M. tuberculosis* cells too. Dormant bacteria are non-dividing but metabolically active. Interestingly, they are always sensitive to the changes in their environment. Since protein turnover is a function of synthesis and degradation, turnover allows us to look at the proteins more closely thereby increasing chances of detecting subtle changes in the proteome of dormant *M. tuberculosis*. This translates to increased identification of novel drug targets for dormant *M. tuberculosis*.

The limitations in studying protein turnover lies in the design of experiments. Protein turnover studies are performed using isotopic labels for two different states. *In vitro* these experiments can be done suitably by labeling the cells at a certain time point before or after the stress. However, to study the turnover of the proteome in an *in vivo* condition, newer methods need to be implemented since it would be difficult to label the pathogen *in vivo*. 

The studies on *M. tuberculosis* need to be augmented by studies on macrophage biology. Protein turnover analysis can also be used to study the phagosomal compartmentalization in the macrophage. Since phagosome assimilation is a dynamic process, knowledge of the cascading events in the maturation of the phagosome might give us an insight into how a pathogen such as *M. tuberculosis* is able to modulate it thereby fuelling research on interfering molecules that may affect the modulation of host phagosomes by *M. tuberculosis*. 
